# Relationships between arm and leg real-life activity and clinical assessments in individuals with disabling spasticity after stroke

**DOI:** 10.3389/fstro.2026.1731911

**Published:** 2026-03-09

**Authors:** Sofi Andersson, Anna Danielsson, Katharina S. Sunnerhagen, Margit Alt Murphy

**Affiliations:** 1 Department of Clinical Neuroscience, Institute of Neuroscience and Physiology, Sahlgrenska Academy, University of Gothenburg, Gothenburg, Sweden; 2 Department of Occupational Therapy and Physiotherapy, Sahlgrenska University Hospital, Gothenburg, Region Västra Götaland, Sweden; 3 Department of Health and Rehabilitation, Institute of Neuroscience and Physiology, Sahlgrenska Academy, University of Gothenburg, Gothenburg, Sweden; 4 Department of Rehabilitation Medicine, Sahlgrenska University Hospital, Gothenburg, Region Västra Götaland, Sweden

**Keywords:** accelerometry motion sensor, activity, functioning, spasticity, stroke

## Abstract

**Background:**

Accelerometer-based measures can provide valuable and objective information about arm and leg use in daily life. This information can be particularly useful in tailoring treatment and rehabilitation in people with disabling spasticity after stroke. To better understand clinical relevance of accelerometer-based measures, this study aimed to determine the strength of relationships between real-life arm and leg activity and a set of clinical assessments encompassing body function and activity domains.

**Methods:**

Thirty-five individuals with disabling spasticity in the chronic stage of stroke (mean age 56.8 ± 8.9 years; 54% female) were included. Real-life activity was measured over 4 days using wrist- and ankle-worn accelerometers. Unilateral arm and leg activity as well as arm/leg ratio were derived from vector magnitude counts per minute. Associations between accelerometer-based measures and clinical assessments of motor function, spasticity, activity capacity, and self-perceived activity performance were analyzed using Spearman's rank-order correlation.

**Results:**

Affected arm and leg real-life activity showed mostly moderate correlations with motor function and activity capacity assessments (ρ = 0.55–0.76), low correlations with spasticity assessments (ρ = −0.32 to −0.43) and high correlations with self-perceived manual and walking performance (ρ = 0.70–0.82). Arm activity ratio showed high correlations (ρ = 0.73–0.83) with motor function, activity capacity, and self-perceived performance. Real-life activity of the non-affected limbs demonstrated predominantly low correlations with clinical assessments.

**Conclusion:**

Accelerometer-based real-life activity measures of the affected arm and leg, along with activity ratios, provide clinically valid information regarding motor function and activity in people with disabling spasticity. Self-reported activity performance questionnaires can be valid tools for clinical practice when accelerometer-based measurements are not readily available.

## Introduction

Spasticity, characterized by heightened muscle tone, involuntary muscle overactivity, and joint stiffness, frequently evolves in people with hemiparesis over the first months of a stroke (Zeng et al., 2021; Opheim et al., 2014). In combination with muscle weakness and impaired motor control, it can limit dexterity and mobility, with further adverse effects on performance in activities of daily living and quality of life (Zeng et al., 2021; Pundik et al., 2019). Post-stroke spasticity prevalence varies over the course of stroke recovery, ranging from 4% during the first weeks to 42.6% in the chronic stage of stroke (Wissel et al., 2013). Severe or disabling spasticity (Modified Ashworth Scale, MAS ≥3) occurs in 2%−15% of stroke survivors with hemiparesis, reaching a peak at 12 months post-stroke (Zeng et al., 2021). Thus, post-stroke spasticity poses a considerable challenge for rehabilitation and significantly increases caregiver burden (Zeng et al., 2021; Wissel et al., 2013).

Spasticity lacks a single, consistent definition (Zeng et al., 2021; Wissel et al., 2013), but in clinical practice, velocity-dependent stretch reflex characterization (Lance, 1980) and a broader conceptualization of “disordered sensorimotor control, resulting from upper motor neuron lesion, presenting as intermittent or sustained involuntary activation of muscles” (Pandyan et al., 2005) are commonly used. Disabling spasticity is commonly operationalized as a functionally limiting condition perceived by individual or caregiver as limiting activities and/or participation (Biering-Soerensen et al., 2021).

In clinical practice, post-stroke spasticity is commonly assessed as muscle resistance during passive movement (e.g., by MAS) and classified within the body function domain according to the International Classification of Functioning, Disability and Health (ICF) (Opheim et al., 2014; Aloraini et al., 2015). The assessment of spasticity provides, however, limited information on a person's activity capacity (what an individual can do in a standardized environment) or performance level (what an individual actually does in their everyday environment) (World Health Organization, 2001; Tarvonen-Schröder et al., 2015). Thus, in people with disabling post-stroke spasticity, an important knowledge gap remains in understanding how clinical assessments of motor function, spasticity and activity, which cover different ICF domains, correlate with an individual's actual real-life activity in everyday life.

Understanding relationships between ICF domains of body functions, activity capacity and activity performance, is essential for several reasons. First, it will provide a more nuanced ground for individually tailored treatment solutions combining non-pharmacological (physiotherapy, occupational therapy) and pharmacological interventions (Biering-Soerensen et al., 2021). Second, understanding the relationships between clinical assessments and real-life activity patterns may reveal whether a transfer to improved activity performance in real-world participation and quality of life has occurred after treatment (Lang et al., 2013).

Patient-reported questionnaires offer insights into real-life performance, but they are susceptible to recall and social desirability bias (Bailey et al., 2015b). Accelerometer-based measurement provides an objective, ecologically valid alternative (Bernaldo de Quirós et al., 2022; Heye et al., 2022), delivering continuous real-life activity data on upper- and lower-limb movement in naturalistic environments (Bailey et al., 2015b; Gebruers et al., 2010; Rand and Eng, 2015). However, when used as single unit on a body segment, such as the wrist or ankle, accelerometers cannot provide information on movement quality (Heye et al., 2022) or differentiate between volitional and non-volitional movement (Heye et al., 2022; Gebruers et al., 2010). Additionally, wrist-worn accelerometers cannot capture more distal movements, such as finger movements (Pohl et al., 2025). More complex setups with multiple accelerometers can enable analyses comparable to camera-based motion capture, but this approach compromises clinical feasibility and is not applicable to continuous monitoring over several days in real-world living environments.

Accelerometer-based activity measures have shown varying degrees of correlation (very high to very low) with different measures of upper and lower limb functioning in people with stroke (Rand and Eng, 2015; Pohl et al., 2025; Uswatte et al., 2006). For example, upper limb motor function, spasticity, and walking ability together explained 77% of variance in real-life activity in people with chronic stroke (Andersson et al., 2021). However, the shared communalities between accelerometer-based measures and established clinical assessments of ICF impairment, activity capacity, and performance warrant further investigation. Despite growing evidence supporting the use of accelerometer-based measurements in rehabilitation (Bernaldo de Quirós et al., 2022; Heye et al., 2022) they are poorly implemented in clinical practice (Lang et al., 2020; Braakhuis et al., 2021). For better clinical implementation more knowledge is needed on sensor placements, output metrics, and how the activity performance data can be used in clinical decision making in specific groups such as people with stroke-induced spasticity.

This study aimed to investigate to what degree accelerometer-based real-life arm and leg activity correlates with motor function, spasticity, activity capacity, and self-perceived activity performance in people with disabling spasticity in chronic stage of stroke.

## Material and methods

### Study design and participants

**Figure 1:**
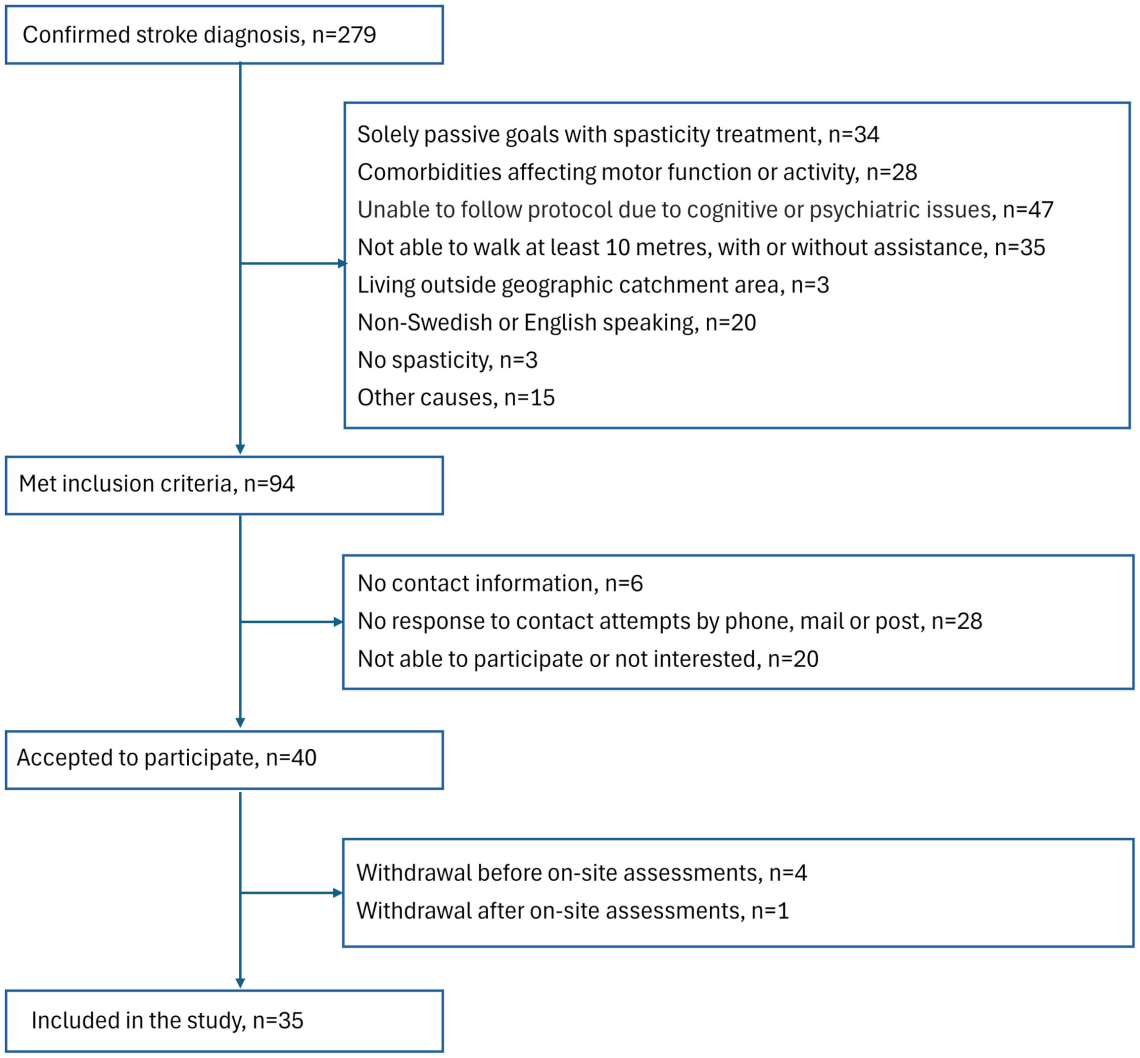
Flowchart of the inclusion process.

In this cross-sectional, observational study, participants were consecutively recruited from Sahlgrenska University Hospital in Gothenburg, Sweden. Patients admitted to the spasticity clinic were screened between November 2021 and November 2023 (Figure 1). Inclusion criteria were: age at least 18 years, confirmed diagnosis of stroke at least 3 months prior, disabling spasticity impacting daily activities (Zeng et al., 2021) and requiring pharmacological treatment (Botulinum toxin A, BoNT-A), ability to walk at least 10 m with or without assistance and ability to communicate in Swedish or English. Exclusion criteria were having solely passive goals with spasticity-reducing treatment (e.g., improve hygiene of the hand, reduce pain), co-morbidity that could affect functioning (e.g., other neurologic disorder, rheumatic disease, or other musculoskeletal impairments), not able to follow the study protocol due to impaired cognitive function, communication, severe psychiatric condition, or abuse.

Ethical approval for the study was obtained from the Swedish Ethical Review Authority (Dnr: 2021-03438). All participants provided written informed consent before participating. This study complies with the Declaration of Helsinki and adheres to the STROBE reporting guidelines for observational research, applying the STROBE checklist for cross-sectional studies (Supplementary material) (von Elm et al., 2007).

The inclusion process is shown in Figure 1.

At study admission, data on age, sex, affected side, diagnosis, and time since stroke were extracted from medical charts. Information about pre-stroke hand dominance and work status was collected through self-report. Leisure-time physical activity was evaluated through interviews using the 4-level Saltin-Grimby Physical Activity Level Scale (SGPALS) (Rödjer et al., 2012). Walking ability was assessed using the Functional Ambulation Categories (FAC), with scores ranging from 0 to 3 indicating dependency and scores from 4 to 5 indicating independence in walking (Mehrholz et al., 2007). Non-motor domains of the Fugl-Meyer Assessment (FMA) were used to assess sensation, range of movement, and pain during passive joint motion. A score less than the maximum score was defined as having an impairment (Fugl-Meyer et al., 1975).

### Real-life activity performance

#### Accelerometer data collection

Four water-resistant 3-axial accelerometers (ActiGraph wGT3X-BT, ActiGraph Corp., Pensacola, FL, USA, 46 x 33 x 15 mm: weight 19 g) were used to measure the three-dimensional acceleration signal (John and Freedson, 2012). The Velcro bands provided by the manufacturer were used to secure the accelerometer units to the wrists, similar to a watch, and on the ankles, directly proximal to the lateral distal malleolus of the tibia (Bernaldo de Quirós et al., 2022; Yang and Hsu, 2010). Participants were instructed to wear the accelerometers for 4 consecutive days (Troiano et al., 2008) while maintaining their usual daily routines. For statistical analysis, a minimum of 20 hours of wake-time wear data per accelerometer was required to ensure sufficient representation of daily activities. Participants were permitted to remove the accelerometers during sleep and activities such as washing, showering, or bathing, as the Velcro straps had a long drying time. Participants recorded accelerometer wear hours and reasons for removal in a wear-time log, which was used solely to verify that the sensors were worn according to protocol (≥20 h of wake-time wear).

#### Accelerometer data processing and analysis

The raw accelerometer data was post-processed using Actilife software (ActiLife^®^, version 6.13.4, ActiGraph UP Corp., Pensacola, FL, USA). Before analysis, the data were visually inspected and cross-checked against wear-time logs to confirm that all accelerometers had been worn for the intended periods. Data from each accelerometer were sampled at 30 Hz (default setting) and aggregated into 10-s epochs. One activity count corresponds to 0.01664 g/count according to manufacturer's proprietary algorithms (Uswatte et al., 2000; Bailey et al., 2015a; Lohse et al., 2025). The standard default ActiGraph filter with bandwidth of 0.25–2.5 Hz (Neishabouri et al., 2022; Brønd and Arvidsson, 2016) was used. No additional filtering or custom windowing procedures were applied. For each epoch, vector magnitude counts were calculated as √(x^2^ + y^2^ + z^2^), where x, y and z represent the activity counts in the vertical, anterior-posterior, and medio-lateral axes, respectively. This approach integrates triaxial signals into a single composite measure of movement magnitude, referred to as vector magnitude counts, providing a standardized overall metric of activity. Only wake-time data were included in the analyses. Vector magnitude counts for each 10-s epoch were averaged over the entire wake-time measurement period and expressed as counts per minute (CPM) to quantify arm and leg activity, with higher CPM values indicating a higher activity level. The activity ratio was calculated as the ratio of CPM between the affected and non-affected limbs (affected/non-affected). This asymmetry measure indicates which limb contributed more activity relative to the other. A value of 1.00 indicates equal accelerometer-derived activity between the extremities, whereas values lower than 1.00 indicate lower activity in the affected limb compared with the non-affected limb.

### Clinical assessments

A battery of standardized clinical scales, validated in stroke populations and recommended for use in stroke trials, was employed (Kwakkel et al., 2017; Alt Murphy et al., 2015). All assessments were administered approximately 1 week before the planned spasticity-reducing treatment by an experienced physiotherapist (SA), who was otherwise not involved in the treatment.

### Body function

The Fugl-Meyer Assessment (FMA) was used for upper and lower extremity motor function. The maximum scores of 66 for the upper extremity and 34 for the lower extremity, respectively, indicate good motor function (Fugl-Meyer et al., 1975). The Modified Ashworth Scale (MAS) was used to assess muscle tone (spasticity) (Bohannon and Smith, 1987). Muscle tone was evaluated for the flexor muscles of the wrist and elbow, as well as the ankle plantar flexors (Ansari et al., 2008). Each muscle group was graded from 0 to 5, with 0 indicating normal muscle tone and 5 indicating that no movement was possible due to rigidity. In this study, the upper-limb score was calculated as the sum of the wrist and elbow muscle group scores, yielding a maximum possible score of 10.

### Activity capacity

The Action Research Arm Test (ARAT) was used to assess arm activity capacity (Lyle, 1981). The ARAT consists of 19 items subdivided into four subscales (grasp, grip, pinch, gross movement). Each item is scored on a 4-point ordinal scale, with a maximum total score of 57 indicating best performance. Comfortable walking speed (m/s) was derived from a 10-meter timed self-paced walking test (Tyson and Connell, 2009), which was used as a measure of leg activity capacity.

### Self-perceived activity performance

The ABILHAND is a measure of manual ability (Penta et al., 1998) that assesses perceived difficulty in performing daily manual tasks, regardless of the strategies used (Penta et al., 2001), through 23 bimanual activities, rated on a 3-level scale (impossible, difficult, easy). The ABILOCO questionnaire measures self-perceived locomotion ability through 13 activities assessing walking performance, rated on a 2-level scale (impossible, possible) (Caty et al., 2008). Both instruments were developed and validated for people with stroke using Rasch modeling, which transforms raw scores into linear logit measures. Logit scores were expressed as percentages, with higher percentages indicating greater perceived ability.

Accelerometer-based measures were classified as ICF activity performance, clinical assessments of Fugl-Meyer Assessment and Modified Ashworth Scale as body function, Action Research Arm Test and 10-m walking test as activity capacity, and ABILHAND and ABILOCO as self-perceived activity performance (World Health Organization, 2001; Tarvonen-Schröder et al., 2015).

### Statistical analysis

For all statistical analyses, IBM SPSS Statistics version 30 (IBM Corporation, Armonk, New York, USA) was used, with the statistical significance level (two-tailed) set at *p* < 0.05. Descriptive statistics were used to summarize demographic and clinical characteristics: means and standard deviations for continuous variables, medians and 25th−75th percentiles (Q1–Q3) for ordinal or skewed continuous variables, and frequencies and percentages for categorical variables. Data distributions were examined visually using histograms and statistically using the Shapiro–Wilk test. Spearman's rank-order correlation (ρ) was used to examine associations between accelerometer-based and clinical measures. Spearman's correlation was chosen because several variables were non-normally distributed, and some measures used ordinal ratings. Ninety-five percent confidence intervals for ρ were obtained using bootstrap resampling (1,000 samples) in IBM SPSS Statistics, version 30. The Wilcoxon signed-rank test was used to determine the differences in counts per minute (CPM) between the affected and non-affected limbs. A one-sample Wilcoxon signed-rank test was conducted to assess whether the activity ratio differed significantly from a perfect activity symmetry (ratio = 1.00). Correlation strength was interpreted using commonly cited guideline thresholds: very low (0.00–0.25), low (0.26–0.49), moderate (0.50–0.69), high (0.70–0.89), and very high (0.90–1.00) (Plichta et al., 2012). These thresholds are intended as guidelines rather than strict cutoffs.

## Results

**Figure 2:**
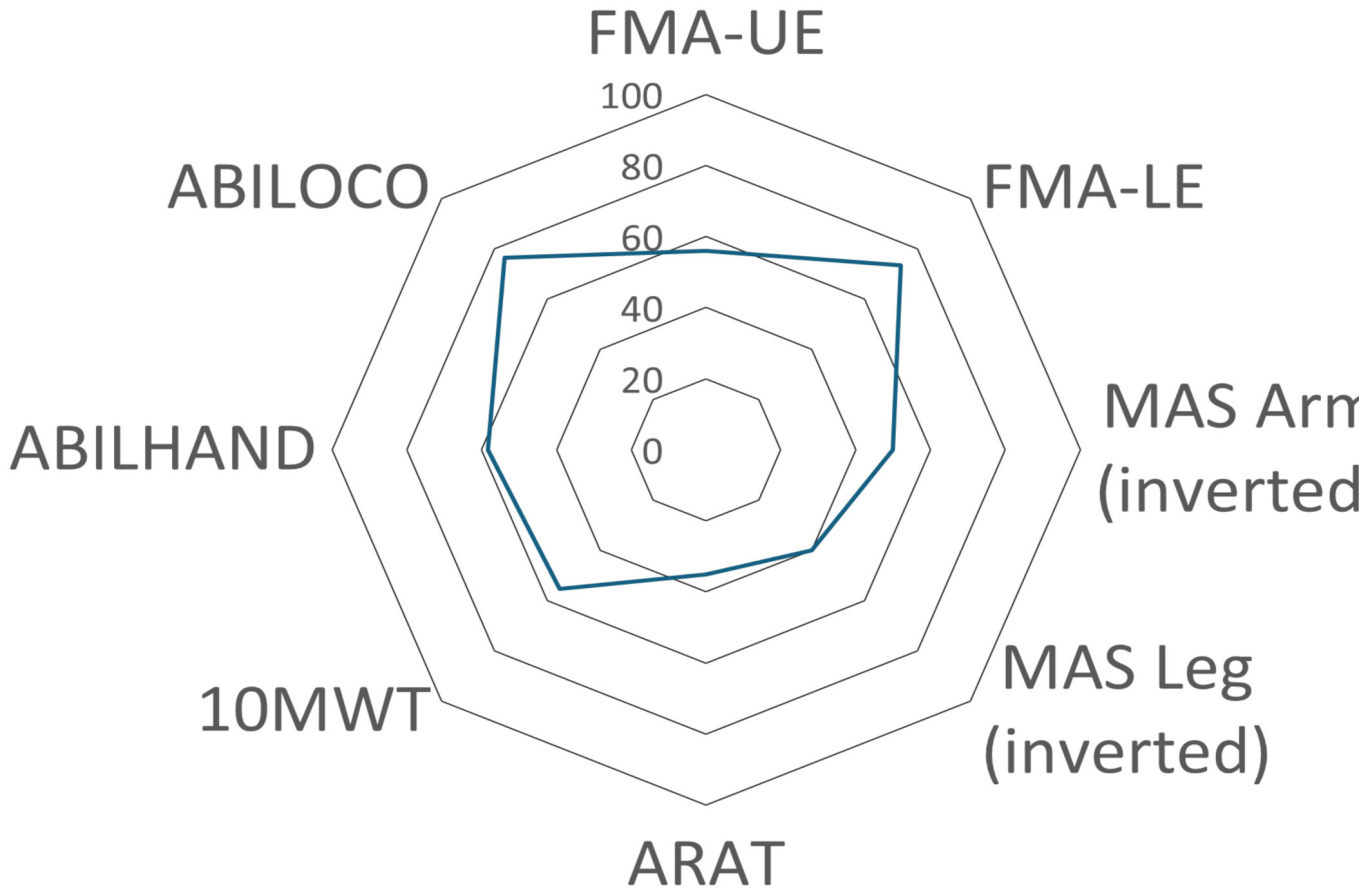
Clinical assessments (*n* = 35) displayed as a percentage of the maximum core. The highest walking speed observed in the study sample (1.57 m/s) was set as 100%. FMA, Fugl-Meyer Assessment; UE, upper extremity; LE, lower extremity; MAS, modified Ashworth Scale; 10MWT, 10-meter walking test; ARAT, Action Research Arm Test.

The demographic and clinical characteristics of the 35 participants are presented in Table 1 and Figure 2. Participants showed considerable variability in age, time since stroke, and motor function (Table 1). This reflects a clinically heterogeneous sample of community-dwelling ambulatory individuals with disabling spasticity treated at a specialized spasticity clinic. The age of the participants ranged between 36 and 71 years (mean, 56.8 years), and 54% (*n* = 19) of the participants were women. Motor function, assessed by FMA, ranged from 6 to 61 points for the upper extremity and from 10 to 31 for the lower extremity. Most participants (80%, *n* = 28) walked independently, while 11% (*n* = 4) required physical support to walk at least 10 meters. A majority (80%, *n* = 28) of the participants showed a significant increase in muscle tone (MAS ≥ 3) in at least one of the tested muscle groups: wrist flexors, elbow flexors, or ankle plantar flexors.

**Table 1:** Demographics and clinical characteristics (*n* = 35).

**Characteristic**	**Total (*n* = 35)**
Age, years, mean (SD)	56.8 (8.9)
Female, *n* (%)	19 (54.3)
Years since stroke onset, median (Q1–Q3)	4.5 (2.0–12.5)
Haemorrhagic stroke, *n* (%)	15 (42.9)
Ischaemic stroke, *n* (%)	20 (57.1)
Right hemiparesis, *n* (%)	16 (45.7)
Right-hand dominant, *n* (%)	31 (88.6)
Left hand dominant or bimanual, *n* (%)	4 (11.4)
Dominant side affected, *n* (%)	13 (37.1)
Impaired sensation UE/LE, *n* (%)	22 (62.9)/23 (65.7)
Decreased ROM UE/LE, *n* (%)	30 (85.7)/33 (94.3)
Joint pain UE/LE, *n* (%)	17 (48.6)/9 (25.7)
Physical activity (SGPALS, 0–4), median (Q1–Q3)	2 (1–2)
FAC (0–5), median (Q1–Q3)	4 (4–5)
Working, *n* (%)	12 (34.3)
**Body function**	
**Upper extremity**	
Motor function (FMA-UE, 0–66), median (Q1–Q3)	37 (17–54)
Spasticity (MAS, 0–10), median (Q1–Q3)	5 (3–6)
**Lower extremity**	
Motor function (FMA-LE, 0–34), median (Q1–Q3)	25 (18–26)
Spasticity (MAS, 0–5), median (Q1–Q3)	3 (2–3)
**Activity capacity**	
ARAT (0–57), median (Q1–Q3)	20 (3–49)
Walking speed (m/s), median (Q1–Q3)	0.87 (0.28–1.04)
**Activity performance**	
ABILHAND (0–100%), mean (SD), *n =* 34	57.8 (12.7)
ABILOCO (0–100%), mean (SD), *n =* 34	73.7 (22.5)

SD, standard deviation; FMA, Fugl-Meyer Assessment; UE, upper extremity; Q1–Q3, 1st and 3rd quartile; ROM, range of motion; LE, lower extremity; MAS, modified Ashworth Scale; ARAT, the Action Research Test; SGPALS, Saltin-Grimby Physical Activity Level Scale; FAC, Functional Ambulation Categories.

*n* = 34 for ABILHAND and ABILOCO; one participant did not complete self-reported activity performance questionnaires.

### Real-life activity performance

**Figure 3:**
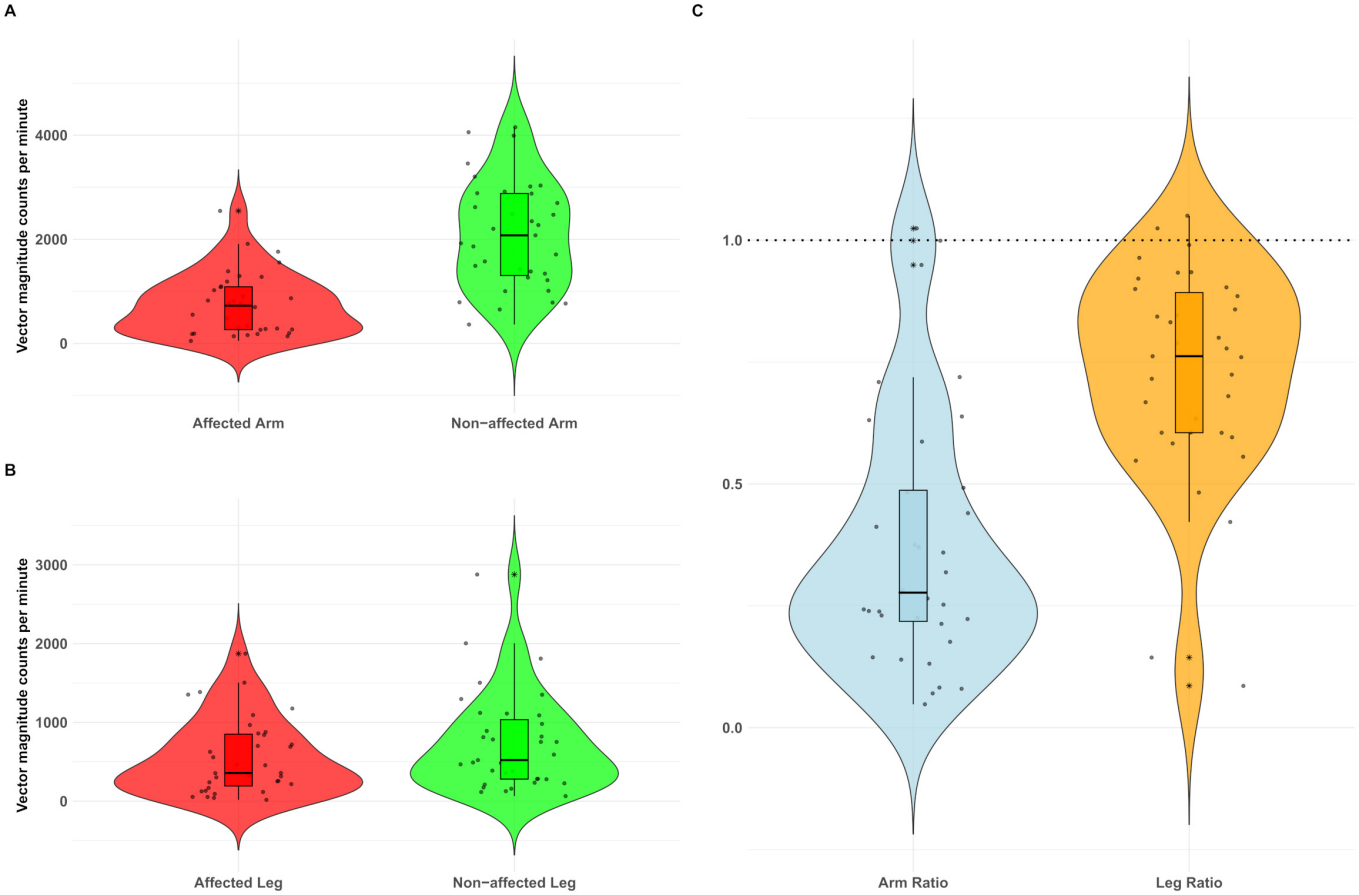
Violin plots showing arm **(A)**– leg **(B)**– and arm and leg ratios **(C)** of real-life activity based on the vector magnitude counts per minute. Each dot represents an individual participant. Box plots within the violins display the median and interquartile range for the study population (*n* = 35). Outliers are marked by asterisks (*) beyond the whiskers of the box plot. In **(C)**– an activity ratio of 1– indicating symmetry between extremities– is represented by a dashed line.

The median wear time of the accelerometers was 46 hours (Q1-Q3: 35–57 h). The median CPM was 725 and 2,076 for the affected and non-affected arms, respectively, and 358 and 522 for the affected and non-affected legs, respectively (Table 2 and Figure 3). Real-life activity was lower in the affected arm (median difference of 1352, z = 5.08, *p* < 0.001) and leg (median difference of 164, z = 4.979, *p* < 0.001) compared to the non-affected side. The arm (median = 0.28) and leg ratios (median = 0.76) were significantly different from the perfect symmetry value of 1.00 (*p* < 0.001) (Table 2 and Figure 3).

**Table 2:** Accelerometer-derived real-life activity expressed as counts per minute (CPM) values shown as mean (SD) and median (Q1–Q3) (*n* = 35).

**Accelerometry measure**	**Mean (SD)**	**Median (Q1–Q3)**
Affected arm	756.8 (594.7)	724.7 (261.7–1,093.1)
Affected leg	558.9 (479.1)	357.6 (168.0–859.6)
Non-affected arm	2,094.6 (1,027.9)	2,076.2 (1,265.8–2,884.8)
Non-affected leg	736.9 (619.9)	521.5 (278.4–1,089.3)
Arm activity ratio	0.36 (0.26)	0.35 (0.21–0.49)
Leg activity ratio	0.76 (0.22)	0.69 (0.61–0.90)

### Real-life activity correlations with clinical assessments

Table 3 presents all pairwise associations between real-life activity measures and clinical assessments. The real-life activity of the affected arm and leg, along with their activity ratios, all demonstrated moderate to high correlations (ρ > 0.50) with clinical assessments of motor function, arm activity capacity, and walking capacity (Table 3). Most correlations with self-perceived activity performance were high (ρ > 0.70). In contrast, spasticity assessments of arm and leg showed predominantly low, negative correlations with activity (ρ = −0.23 to −0.54). The arm activity ratio showed a high correlation (ρ > 0.70) with all clinical assessments, except for the spasticity assessment. Real-life activity of the non-affected arm showed only low correlations (ρ ≤ 0.31) with clinical assessments, while correlations between the non-affected leg activity and clinical assessments of walking capacity, as well as self-perceived manual and walking performance, were moderate to high (ρ ≤ 0.77).

**Table 3:** Spearman's rank-order correlations (ρ) between accelerometer-based real-life activity measures and clinical assessments, categorized by ICF function and activity domains (*n* = 35).

**Accel data**	Body function				Activity capacity		Self-perceived activity performance	
	**FMA-UE**	**FMA-LE**	**MAS-UE**	**MAS-LE**	**ARAT**	**10mWT**	**ABILHAND**	**ABILOCO**
Aff arm	0.64^**^ (0.41, 0.81)	0.57^**^ (0.29, 0.76)	−0.35 (−0.61, −0.00)	−0.41^**^ (−0.64, −0.09)	0.58^**^ (0.28, 0.79)	0.62^**^ (0.30, 0.83)	0.75^**^ (0.58, 0.85)	0.81^**^ (0.65, 0.90)
Aff leg	0.61^**^ (0.34, 0.79)	0.58^**^ (0.33, 0.77)	−0.32 (−0.59, 0.03)	−0.43^**^ (−0.68, −0.12)	0.55^**^ (0.27, 0.77)	0.76^**^ (0.55, 0.88)	0.70^**^ (0.46, 0.84)	0.82^**^ (0.64, 0.90)
Non-aff arm	0.039 (−0.28, 0.35)	−0.018 (−0.35, 0.35)	0.088 (−0.22, 0.37)	−0.25 (−0.55, 0.06)	−0.052 (−0.40, 0.31)	0.074 (−0.29, 0.44)	0.22 (−0.03, 0.52)	0.31 (−0.03, 0.59)
Non-aff leg	0.49^**^ (0.19, 0.71)	0.47^**^ (0.15, 0.71)	−0.23 (−0.54, 0.13)	−0.40^*^ (−0.67, −0.45)	0.42^*^ (0.10, 0.67)	0.67^**^ (0.44, 0.81)	0.62^**^ (0.34, 0.79)	0.77^**^ (0.55, 0.89)
Arm ratio	0.80^**^ (0.58, 0.90)	0.73^**^ (0.51, 0.85)	−0.48^*^ (−0.74, −0.15)	−0.33 (−0.61, −0.20)	0.77^**^ (0.51, 0.91)	0.80^**^ (0.59, 0.91)	0.73^**^ (0.54, 0.86)	0.83^**^ (0.65, 0.91)
Leg ratio	0.62^**^ (0.36, 0.79)	0.61^**^ (0.29, 0.81)	−0.54^**^ (−0.78, −0.25)	−0.24 (−0.52, 0.10)	0.60^**^ (0.32, 0.79)	0.61^**^ (0.29, 0.84)	0.65^**^ (0.40, 0.81)	0.51^**^ (0.18, 0.77)

^*^p < 0.05.

^**^p < 0.01.

Strength of correlation: 0.00–0.25 (very low), 0.26–0.49 (low), 0.50–0.69 (moderate), 0.70–0.89 (high), 0.90–1.00 (very high).

Values are Spearman's ρ (95% confidence interval).

Accel, accelerometry; Aff, affected; Non-aff, non-affected; UE, upper extremity; LE, lower extremity; FMA, Fugl-Meyer Assessment; MAS, Modified Ashworth Scale; ARAT, Action Research Arm Test; 10mWT, 10 m walking test.

## Discussion

This cross-sectional study demonstrated moderate to high correlations between real-life arm and leg activity and clinical assessments of motor function, activity capacity, and self-perceived activity performance in individuals with disabling spasticity during the chronic stage of stroke. In contrast, correlations between real-life activity and spasticity measures were predominantly low. Self-perceived activity measures showed high correlations with real-life activity, and correlations were generally higher for unilateral real-life activity of the affected arm and leg than for the non-affected side. These findings provide much-needed information for a sparsely studied patient group: individuals with stroke-induced disabling spasticity.

Motor function and activity capacity demonstrated higher associations with real-life activity than spasticity in the current study, a finding that aligns with prior research (Pundik et al., 2019; Sommerfeld et al., 2012). In our cohort, correlations between real-life activity and motor function or activity capacity were moderate to high (ρ = 0.55–0.80), whereas correlations with spasticity were predominantly low (ρ = −0.24 to −0.54). This pattern is supported by a study in subacute stroke, where upper limb spasticity assessed by MAS explained only 6% of variance in real-life arm activity, whereas motor function explained 29% in subacute stroke (Andersson et al., 2021). Similarly, another study during the first 3 months post-stroke showed low correlation (ρ = −0.31) between elbow flexor spasticity and arm use (Lang et al., 2007). These findings suggest that accelerometer-based measures predominantly capture aspects of motor function and activity rather than spasticity, and that MAS provides limited information about real-life activity. Consistent with findings in patient cohorts with post-stroke spasticity, real-life activity has shown moderate to high correlations (ρ = 0.54–0.78) with both motor function and activity capacity measures in stroke populations, for which spasticity prevalence or severity was not specified (Rand and Eng, 2015; Pohl et al., 2025; Uswatte et al., 2006; Narai et al., 2016; Demers et al., 2024; Gebruers et al., 2008, 2014).

The low associations between MAS and real-life activity may reflect fundamental differences in measurement approaches and the ICF domains they address. The MAS is widely adopted for its clinical simplicity and accessibility (Haugh et al., 2006), and assesses ICF body function through combined biomechanical and neural contributions to passive stretch resistance at rest in a standardized and controlled environment. In contrast, accelerometer-based real-life activity measures ICF activity performance by actual voluntary and involuntary movements in a naturalistic, uncontrolled context (Heye et al., 2022; Bailey et al., 2015a). These represent contextually distinct constructs: MAS captures impairment-level assessment of spasticity, whereas accelerometry measures activity-focused arm and leg use in daily life. This distinction likely contributes to the low correlations between the two measures.

Accelerometry-based and self-perceived measures both assess the ICF activity performance, which can explain the high correlations found between these measures in the current study. Correlations between objective real-life activity measures and self-perceived activity, assessed with ABILOCO and ABILHAND were high (ρ = 0.70–0.82). This was consistent with prior research, which has shown high (*r* = 0.84) (Narai et al., 2016) and moderate correlations (*r* = 0.52–0.66) (Uswatte et al., 2006; Demers et al., 2024) between real-life arm use ratio and self-reported arm use, whereas the real-life use of affected arm showed moderate (*r* = 0.58) (Narai et al., 2016) and low correlations (*r* = 0.38–0.41) with self-reported arm use (Uswatte et al., 2006). In these studies, the Motor Activity Log (MAL), REACH tool (Uswatte et al., 2006; Narai et al., 2016; Demers et al., 2024) and Actual Amount of Use Test (AAUT) (Uswatte et al., 2006) were used to assess self-reported arm use. These variations in reported correlations may be caused by the differences in instruments used; the ABILHAND, as used in the current study, comprises predominantly bimanual daily activities, whereas the focus of MAL and AAUT is on unilateral arm activity. The REACH instrument, employed by Demers et al. (2024) assesses both unilateral and bimanual activities. To our knowledge, no previous study has specifically examined relationships between self-reported and real-life accelerometer-based leg activity. Taken together, our findings indicate that self-reported activity performance aligns substantially with actual real-life arm and leg use. This implies that ABILHAND and ABILOCO may serve as time-efficient, easy-to-use proxies for assessing activity performance in clinical settings.

Real-life activity of the non-affected arm and leg showed low correlation with clinical measures in the current study. This finding is consistent with recent findings in chronic stroke (Pohl et al., 2025), reporting low correlations (ρ = 0.31–0.35) between non-affected arm activity and arm motor function (FMA), activity capacity (ARAT), and self-reported arm use (MAL Amount of Use) in chronic stroke. Collectively, these low correlations suggest that measuring the real-life activity of the non-affected side alone has limited clinical value (Gebruers et al., 2010; Sánchez-Sánchez et al., 2021). Interestingly, accelerometer-based non-affected leg activity showed moderate-to-high correlations (ρ = 0.62–0.77) with walking speed and self-perceived activity (ABILHAND, ABILOCO). This finding likely reflects the bilateral nature of walking, in which motor impairments in the affected leg also limit the use of non-affected leg (Peters et al., 2021). This underscores that activity of the non-affected leg can provide meaningful information about walking.

Accelerometer placement is critical for interpretation of the activity data. Considering the results of the current study, preferably accelerometers should be placed on the affected arm and leg when the primary focus is on quantification of movements of the affected side. However, considering the well-established evidence for the arm activity ratio as a measure of symmetry, bilateral arm placement should always be considered in people with stroke (Gebruers et al., 2010; Pohl et al., 2025; Gagné-Pelletier et al., 2025).

Clinical value of leg ratio measures is less studied. Previous work in subacute stroke showed that both arm and leg ratios differed significantly from healthy controls, but the arm ratio showed a larger asymmetry than the leg ratio (Alt Murphy et al., 2019). In line with our present findings, this suggests that the arm activity ratio may be a more sensitive indicator of asymmetry than the leg activity ratio. Furthermore, the higher correlations found between arm ratio and clinical assessments in the current study suggest their potential value in clinical practice, particularly in resource-limited settings where only a limited number of accelerometers are available or clinically feasible. An advantage of ratio-based measures over unilateral measures is that they are less sensitive to non-volitional activity (Bailey et al., 2015b; Heye et al., 2022). This is in line with recent work demonstrating good concurrent validity between arm use ratios and upper-limb motor function (FM-UE, ARAT) in large heterogeneous stroke cohorts (Lohse et al., 2025).

### Methodological strengths and limitations

This study uniquely addresses a poorly characterized population by investigating real-life activity in individuals with disabling spasticity in the chronic stage of stroke. All individuals with chronic stroke referred to the specialist spasticity management team were screened for eligibility, thereby strengthening external validity and demonstrating that the sample reflects clinical practice within this care pathway. A comprehensive battery of validated clinical assessments was used, covering motor function, activity capacity, and self-perceived performance in accordance with the ICF framework. This approach allows for a more nuanced interpretation of real-life activity in terms of functioning. The Modified Ashworth Scale (MAS) was selected for spasticity assessment despite the availability of more sophisticated alternatives (e.g., Tardieu Scale), based on its widespread clinical adoption (Opheim et al., 2014; Biering-Soerensen et al., 2021; Aloraini et al., 2015; Haugh et al., 2006) and demonstrated reliability in the muscle groups assessed (Bohannon and Smith, 1987; Ansari et al., 2008), which enables comparisons with broader stroke literature.

A notable methodological strength was the absence of data loss or technical failures, achieved through experienced personnel and support for participants as needed. While the focus of the current study was on correlations between accelerometry and clinical assessments at different ICF domains, comparative data from stroke populations without disabling spasticity would have added value for the interpretation of the results.

The relatively small sample size (*n* = 35) constrains statistical power, the precision of correlation estimates, and the ability to control for potential confounders (age, time since stroke, impairment severity). Recruitment from a single specialized spasticity clinic, limits generalizability to other regions with different stroke care pathways and referral criteria to spasticity treatment. Sample heterogeneity in age, time since stroke, and motor function likely increased sample variability and reduced correlation precision but also enable generalizability of our findings to similar clinical populations commonly treated in specialized spasticity services. Wear-time varied across participants (35–57 h), primarily due to differences in wake-time duration, which might have influenced precision. The cross-sectional single-group study design does not allow any between-group, causal or predictive inference analyses (Hinkle et al., 2003).

## Conclusion

Real-life activity, derived from accelerometer measurements of the affected arm and leg, showed moderate-to-high correlations with motor function and activity capacity assessments but low correlations with spasticity severity in individuals with disabling spasticity in the chronic stage of stroke. Self-perceived activity performance showed high correlations with real-life activity, providing a potential, easily accessible tool for assessment of real-life activity performance in clinical settings. Accelerometer placement on the affected side should be prioritized over the unaffected side, and arm ratio measure may provide a better symmetry index than the leg ratio. The findings of the study provide novel data on a poorly studied group, individuals with disabling post-stroke spasticity, but future studies are needed to improve further the clinical utility of accelerometer-based real-life activity monitoring in routine clinical practice.

## Data Availability

The datasets presented in this article are not readily available because data from this study are available from the corresponding author upon reasonable request. According to Swedish Regulations, an application to and approval by the Swedish Ethical Review Authority are required for data use. Requests to access the datasets should be directed to sofi.andersson@neuro.gu.se.
